# Two Palatal Roots in Maxillary First Molar, a Rare Entity: Report of Two Cases

**DOI:** 10.30476/dentjods.2023.98649.2101

**Published:** 2024-06-01

**Authors:** Vineeta Nikhil, Padmanabh Jha, Preeti Mishra, Sonal Sahu, Abhishek Bhargava

**Affiliations:** 1 Conservative Dentistry and Endodontics, Subharti Dental College and Hospital, Swami Vivekanand Subharti University, Meerut, India

**Keywords:** Anatomical variations, Extra roots, Endodontic failure, Maxillary molar, Palatal root, Root canal therapy

## Abstract

An in-depth understanding of the anatomical variations of maxillary molars is essential for endodontic success. Unlike the maxillary second molars, the presence of a second palatal root is uncommon in the first maxillary molar. This case report describes two cases of non-surgical management of maxillary molars with extra palatal roots. Careful clinical examination, knowledge of the internal anatomy, and the use of advanced radiographic modalities like cone beam computed tomography (CBCT) can reveal the presence of variations in the internal and external anatomy of any tooth. Therefore, for nonsurgical as well as surgical management clinicians should always watch out for any deviations in a tooth and utilize all the available tools to diagnose and manage them successfully.

## Introduction

Endodontic treatment is aimed to completely eliminate microorganisms from the root canal space and to prevent its reinfection. Root canal treatments often fail due to the presence of extra canals or extra roots that are left untreated [ 1 ]. The wide anatomical variations encountered in molars often present a challenge for endodontists; therefore, a thorough knowledge of such variations, aberrations, and abnormalities is an important pre-requisite to endodontic treatment [ 2 ]. 

Usually maxillary molars have three roots and their corresponding three canals namely, mesiobuccal, distobuccal, and palatal. However, Yadav *et al*. [ 3 ] reported maxillary molar with seven root canals. Maxillary first molars with single root, one, and two canals as well as with two roots and two canals were reported by Janani *et al*. [ 4 ] in their report of three cases. A second mesiobuccal canal is a frequent finding in maxillary first molars with a prevalence ranging from (48% to 97.6 %) [ 5 ], in comparison to maxillary second molars (29.8% to 69%) [ 6 - 10 ]. Additionally single palatal canal, two palatal canals with two separate orifice and two separate foramina or one palatal canal with two separate foramina in single palatal root have also been reported [ 11 ]. The reported incidence of an extra canal in the palatal root of maxillary molars is 2–5.1% [ 12 ]. 

Maxillary molars with two separate palatal roots have also been reported. Fakhari *et al*. [ 13 ] and Nabavizadeh *et al*. [ 14 ] reported presence of two separate palatal roots in the maxillary second molars. However, the prevalence of a second palatal root is very rare and in the maxillary second molars it is found to be less than or equal to 1.4% [ 15 ]. Asghari *et al*. [ 16 ] reported management of maxillary first molar with two separate palatal roots. The incidence of maxillary first molars with an extra palatal root is unknown in the literature. 

Christie *et al*. [ 1 ] classified maxillary molars with two palatal roots into three types on the basis of radiographic appearance: *Type I*; the palatal roots are more divergent and long compare to the buccal roots, *Type II*; the buccal and palatal roots are relatively shorter, have blunt apices and are parallel to each other and gives radiographically appearance of only two roots mesial and distal, *Type III*; both the palatal and the mesiobuccal canals are engaged in a web of root dentin and the distobuccal root diverges to remain separate. 

Cone beam computed tomography (CBCT) has an edge over intraoral imaging as it provides information of all the three dimensions of maxillofacial structures. This feature enables the endodontist to visualize the complex tooth morphology and root canal system without any anatomic obstruction. Therefore, CBCT not only aids in diagnosing the disease but also customizing the treatment plan according to clinical situation [ 17 ]. 

This case report compiles management of two cases of maxillary first molars with the unusual presence of extra palatal roots that were confirmed with CBCT and intraoral periapical radiograph.

## Case Presentation

### Case 1

**Figure 1:**
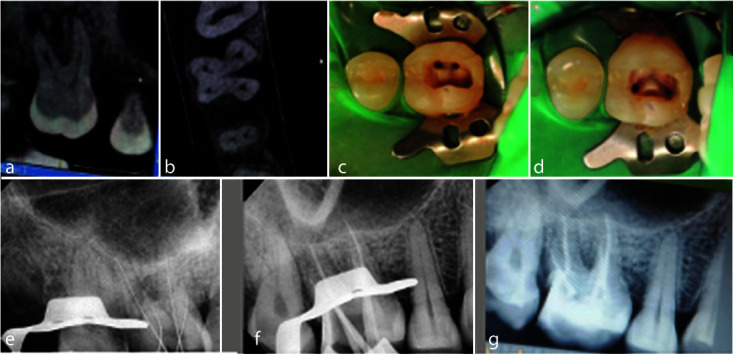
**a-b:** Pre-operative cone beam computed tomography (CBCT) images, **c:** Mesiobuccal and distobuccal canal orifices, **d:** Two separate palatal orifices, **e:** Working length verification with radiograph, **f:** Mastercone selection. **g:** Obturated root canals and post-endodontic composite restoration

A 45-year-old female reported with pain in the upper right posterior tooth. Medical history and dental history were non-significant and there was no history of previous extraction in the maxillary right quadrant. Patient’s oral hygiene was poor and on clinical examination, proximal caries was observed on the distal surface of maxillary right first molar (tooth number 16). No sign of extraoral or intraoral swelling was present. The tooth was tender on percussion and exhibited prolonged sensitivity to cold test compared to the control tooth. The electric pulp test yielded a positive response. Radiographic examination revealed distal radiolucency involving enamel, dentin, and pulp. An extra palatal root was suspected on exposing the tooth to multiple angles for intraoral periapical radiographs, which was confirmed with preoperative CBCT. The tooth morphology was identified as *Type I* according to Christie *et al*. classification [ 1 ]. The final diagnosis was symptomatic irreversible pulpitis with symptomatic apical periodontitis. The treatment plan comprised oral prophylaxis followed by endodontic treatment and a full coverage restoration. Informed consent was taken for the treatment and for publication of the case (Figure 1).

2% lignocaine with epinephrine 1:1,00,000 (Lignox, Indoco, India) was administered to anaesthetized tooth number 16 with posterior superior alveolar block and local infiltration. Anesthesia was confirmed with electric pulp tester. Rubber dam was applied and caries was excavated with a round diamond point (Mani, Inc Japan), the pre-endodontic build-up was done with composite before access cavity preparation. Under 3.5× magnification loupe (Zumax medical Co, China), the three canals mesiobuccal, distobuccal, and palatal canals 

were located. On exploration with DG 16 explorer, a catch was felt slightly distal to the main palatal canal. The presence of the extra palatal canal was confirmed by scouting with a #10K file (Dentsply Maillefer, Switzerland). Patency was established in all four canals and working length was determined with an electronic apex locator (Coltene, Altstätten, Switzerland) and confirmed radiographically. Canals were prepared using the ProTa-per Gold system (Dentsply Maillefer, Ballaigues, Switzerland) to a size of F2 and throughout preparation; canals were irrigated with 3% sodium hypochlorite (CanalPro, Coltene Whaledent). Canals were finally irrigated with normal saline and calcium hydroxide (Prevest Den-Pro, India) dressing was placed for two weeks as intra canal medicament. The pulp canal orifices were blocked with Teflon and the access opening was sealed with temporary filling material (Orafil-G, Prevest DenPro, India). Patient was referred to the Department of Periodontology for oral prophylaxis and education and motivation for maintenance of oral hygiene. On second appointment, as the patient was completely asymptomatic, canals were irrigated with 17% EDTA solution (Prevest DenPro, India), agitated with EndoActivator (Dentsply Maillefer) to remove intra canal medicament, and dried with absorbent paper points (Dentsply Maillefer). Single cone obturation of all four root canals was done using a bioceramic sealer Bioroot RCS, (Septodont, St. Maurdes Fossés, France) and gutta percha (Dentsply Maillefer). The post-endodontic restoration was done with a nanohybrid composite (Ivoclar Vivadent, Schaan, Liechtenstein), followed by a full-coverage crown. 

### Case 2

**Figure 2:**
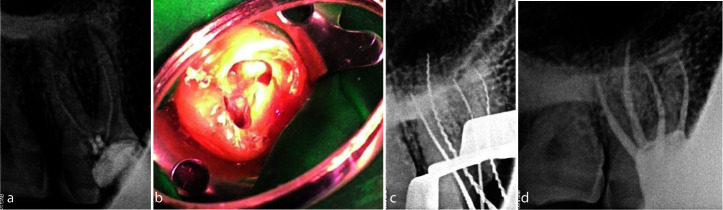
**a:** Pre-operative intraoral periapical radiograph, **b:** Access opening showing two palatal canal orifices, **c:** Working length radiograph, **d:** Post obturation intraoral periapical radiograph of tooth #16

A 35-year-old female with no systemic disease reported with a history of pain in relation to the upper right posterior region. History of dental treatment revealed root canal treatment in the upper right first molar, around 1 year back. Clinical inspection revealed dislodged restoration and secondary caries in respect to tooth number 16. Tenderness on palpation was positive with no signs of intraoral or extraoral swelling and draining sinus. Oral hygiene was fair. Radiographic examination revealed underextended and underfilled obturation in mesiobuccal and distobuccal root canals. To redress the unsatisfactory endodontic treatment and relieve the symptoms of the patient, the retreatment of tooth number 16 was planned for this case. The patient was informed about the line of treatment for the concerned tooth and consent was obtained for treatment (Figure 2).

Tooth number 16 was anesthetized with 2% lignocaine with epinephrine 1:1,00,000 (Lignox, Indoco, India) with posterior superior alveolar block and local infiltration. Isolation was achieved with rubber dam. The remaining old restorative material was removed and caries excavation was performed with a round diamond point (Mani, Inc Japan) in a high-speed handpiece. The pulp chamber was cleaned and gutta-percha filled orifices were located using 3.5 x loop (Zumax medical Co, China). Using H files (Mani, Inc Japan), the gutta-percha was eliminated from the mesiobuccal, distobuccal, and palatal canals. Upon removing the obturating material, the pulpal floor was cleaned to facilitate a better understanding of the dentinal map. While following the dentinal map, an extra canal was suspected near the palatal canal. DG 16 and small K files of sizes #6 and 8 (Dentsply Maillefer, Switzerland) were used to get a sticky catch and negotiate the extra palatal canal. Patency was achieved and working length was determined for all four canals using an electronic apex locator (Coltene, Altstätten, Switzerland). The extra palatal root was confirmed with radiographs revealing *Type I* morphology according to Christie *et al*. classification [ 1 ]. Biomechanical preparation was done using the ProTaper Gold system (Dentsply Maillefer, Ballaigues, Switzerland) to a size of F2 for mesiobuccal and distobuccal canals and F3 for the palatal canal. Throughout preparation, canals were irrigated with 3% sodium hypochlorite (CanalPro, Coltene Whaledent). Aqueous mixture of calcium hydroxide powder (Prevest DenPro, India) was placed for 14 days. The pulp canal orifices were blocked with Teflon and the access opening was sealed with temporary filling material (Orafil-G, Prevest DenPro, India). With the resolution of the patient’s symptoms after two weeks, canals were obturated with a single cone obturation technique using a bioceramic sealer (Bioroot RCS, (Septodont, St. Maurdes Fossés, France) and gutta perc-ha (Dentsply Maillefer). The final restoration was done with a nanohybrid composite (Ivoclar Vivadent, Schaan, Liechtenstein). Informed consent for publication of the case was obtained.

## Discussion

The possibilities of encountering morphological variations in endodontic practice are endless. Similarly, maxillary molars also present challenges during treatment because of wide variety of variations seen in them. Therefore, one of the essential requirements of successful root canal treatment is thorough understanding of root canal system. There are several ways to detect hidden or missed canals [ 18 ]. Some clinical techniques include the use of endodontic explorers like DG 16, champagne bubble test using sodium hypochlorite, red line test, white line test, and reading the dentinal map. The use of magniﬁcation has emerged out as exceptional tool that aid in identifying and managing extra canals [ 18 ]. Tuculina *et al*. [ 19 ] in their study reported that through the use of dental microscope, additional canals were identified in the case of 32.25% of the second maxillary molars examined. 

To diagnose variations in morphology, thorough reading of preoperative radiographs is must. Since a two-dimensional radiographs have limitations of superimposition and distortion, utilization of 3 dimensional CBCT is more useful in understanding root canal morphology particularly in rare and doubtful cases [ 20 ]. In the first case, the use of CBCT served as an important pre-diagnostic tool. The use of CBCT in the identification of extra palatal roots has been reported previously [ 21 - 23 ]. In the second case of retreatment, the knowledge of the root canal anatomy, Krasner and Rankow’s law of color change [ 24 ], and the law of orifice location helped in determining the extra canal of the extra palatal root. Additional use of magnification greatly aided in the precise location and management. Both cases fall in *Type I* category of Christie *et al*.’s classification [ 1 ]. 

In summary, present report involved the successful retreatment and nonsurgical management of the maxillary first molar with a second palatal root. Prudent preoperative radiographic analysis and examination of pulp chamber floor helped to discover this anatomical variation. Information about variations should be disclosed to the patients while obtaining the consent for the treatment from the patients.

## Conclusion

Clinicians should be aware of uncommon root morphologies and pulp canal configurations. Prudent analysis of preoperative radiographs shot at different horizontal angles, use of 3 dimensional radiography, applications of the principles of root canal locations, and use of magnification tools are essential to find out any deviation in the tooth and root canal system configuration for successful outcome of surgical or nonsurgical treatment.
